# The prevalence and treatment pattern of clinically diagnosed pelvic organ prolapse: a Korean National Health Insurance Database-based cross-sectional study 2009–2015

**DOI:** 10.1038/s41598-018-19692-5

**Published:** 2018-01-22

**Authors:** Jin-Sung Yuk, Jung Hun Lee, Jun-Young Hur, Jung-Ho Shin

**Affiliations:** 1 Department of Obstetrics and Gynecology, College of Medicine, Gyeongsang National University, Gyeongsang National University Changwon Hospital, Changwon-si, Gyeongsangnam-do 51472 Republic of Korea; 2 Department of Obstetrics and Gynecology, Korea University College of Medicine, Seoul, 08308 Republic of Korea

## Abstract

The study aim was to evaluate the prevalence of pelvic organ prolapse using claim data of South Korea and to evaluate treatment patterns. The Korea National Health Insurance Corporation pay medical costs for most diseases. This study used Health Insurance Review & Assessment Service-National Inpatient Sample (HIRA-NIS) 2009–2015. Pelvic organ prolapse was defined by diagnostic code (N81.x). Of the approximately 4.5 million women included in HIRA-NIS 2009–2015, 10,305 women were selected as having pelvic organ prolapse, and the mean age of the pelvic organ prolapse group was 63.9 ± 0.2 years. The prevalence of pelvic organ prolapse was 180 ± 4 per 100,000 population in women older than 50 years old. In logistic regression analysis, constipation increased the prevalence of all pelvic organ prolapse (odds ratio, 4.04; 95% confidence interval, 3.52–4.63; P < 0.01). The number of women requiring pessary only and surgery only were 26 ± 2 per 100,000 population and 89 ± 1 per 100,000 population, respectively, for women over 50 years of age. The prevalence of pelvic organ prolapse was quite lower than in previous studies. Surgery peaked at approximately 70 years of age. Pessary increased dramatically among women after the age of 65.

## Introduction

Pelvic organ prolapse (POP) is a disease in which one or more of the female pelvic organs, such as the bladder, uterus, vaginal cuff, rectum and intestine, descend through the vagina^1,2^.

POP is related with various symptoms, such as urinary incontinence, voiding dysfunction, frequency, dyschezia, pelvic heaviness, prolapse sensation, vaginal pain and low back pain^3,4^.

There are a variety of treatments about POP, however the subjective symptoms of the patient are important because the decision to treat POP depends on the discomfort of the patient rather than severity as assessed by physical examination^2^.

The prevalence of POP in previous studies was 2.9–41.1%^5–8^. However, it is difficult to obtain a consistent prevalence because defining POP diagnosis (symptoms, physical examination, and surgery) differs according to the study^5–9^. If symptoms are mild, pelvic floor muscle exercise (PFME) may be sufficient without treatment by a doctor. Even if POP is diagnosed through physical examination, treatment may not be necessary if no symptoms are present^1,10^. Therefore, it is clinically more important to confirm whether a patient visited the clinic due to uncomfortable feelings. It is also clinically important to identify treatments (surgery and pessary) that require the help of a doctor. This study using claim data is appropriate for this purpose because claim data include clinic visit records, diagnosis codes and treatment codes. However, studies of this nature are limited.

The primary purpose of this study was to evaluate the prevalence and risk factors of POP using claim data from South Korea. The second purpose was to evaluate treatment patterns, such as pessary and surgery, in POP patients.

## Results

**Figure 1:**
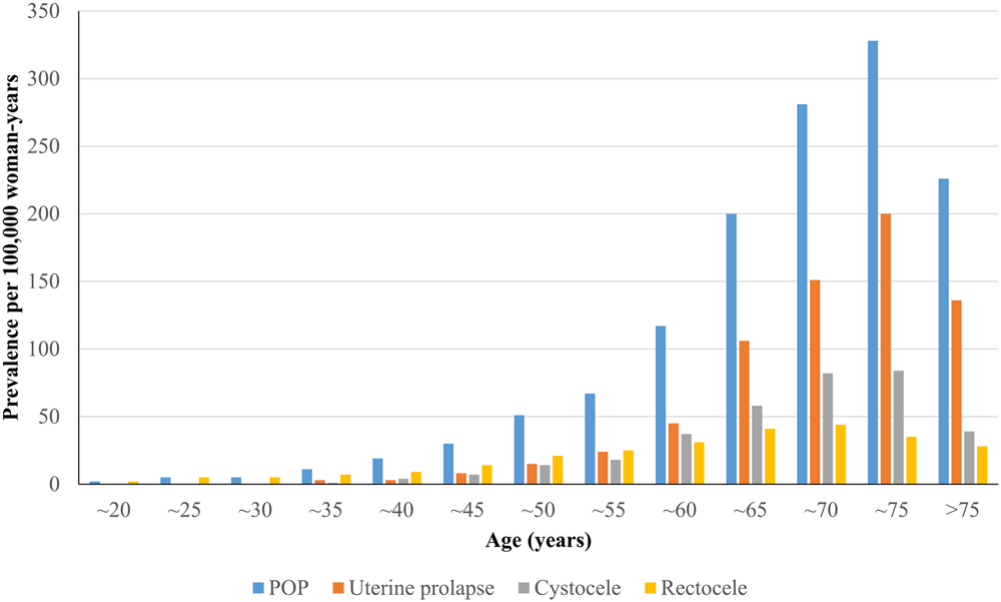
The change in prevalence according to age increments in HIRA-NIS 2009–2015. HIRA-NIS: The Health Insurance Review & Assessment Service-National Inpatient Sample, POP: Pelvic Organ Prolapse.

Of the approximately 8.3 million samples of HIRA-NIS 2009–2015, data from 4,476,495 women were extracted. Of these, we selected 10,305 women with POP, and the mean age of the POP group was 63.9 ± 0.2 years. Of the POP patients, 8,708 were over 50 years old, and the mean age was 67.5 ± 0.2 years (Table 1). The prevalence of POP was 71 ± 1 per 100,000 population at all ages and 180 ± 4 per 100,000 population among women greater than 50 years old. The prevalence of uterine prolapse, cystocele, and rectocele was 35 ± 1 per 100,000 population, 18 ± 1 per 100,000 population, and 16 ± 1 per 100,000 population among all ages, respectively, and 95 ± 3 per 100,000 population, 47 ± 2 per 100,000 population, and 33 ± 1 per 100,000 population among women greater than 50 years of age, respectively. POP, uterine prolapse and cystocele were most commonly observed in women in their early 70 s, and rectocele was most common in women in their late 60 s (Fig. 1). Among the total cases of POPs, uterine prolapse (49.9%) was the most common followed by cystocele (26.1%), rectocele (23.0%), enterocele (0.5%) and urethrocele (0.4%).

**Table 1:** Risk factors of pelvic organ prolapse patients in HIRA-NIS 2009–2015.

	Women of all ages			Women over 50 years of age		
	Women Without POP Diagnostic Codes	POP	P-value	Women Without POP Diagnostic Codes	POP	P-value
	(N = 4,466,190)	(N = 10,305)		(N = 1,928,741)	(N = 8,708)	
Age	40.0 ± 0.0	63.9 ± 0.2	<0.01	63.8 ± 0.0	67.5 ± 0.2	<0.01
Low SES	243,148	616	0.02	165,180	555	<0.01
COPD	32,633	118	<0.01	30,737	116	0.06
Constipation	104,652	875	<0.01	64,252	672	<0.01
Urethrocele	0	41	<0.01	0	27	<0.01
Cystocele	0	3,033	<0.01	0	2,621	
Uterine prolapse	0	5,027	0	0	4,710	0
Vaginal enterocele	0	69	<0.01	0	41	<0.01
Rectocele	0	2,496	<0.01	0	1,672	<0.01
Other POP	0	375	<0.01	0	331	<0.01
Unspecified POP	0	783	<0.01	0	743	<0.01

COPD: Chronic Obstructive Pulmonary Disease, HIRA-NIS: The Health Insurance Review & Assessment Service-National Inpatient Sample, POP: Pelvic Organ Prolapse, SES: Socioeconomic status.

**Figure 2:**
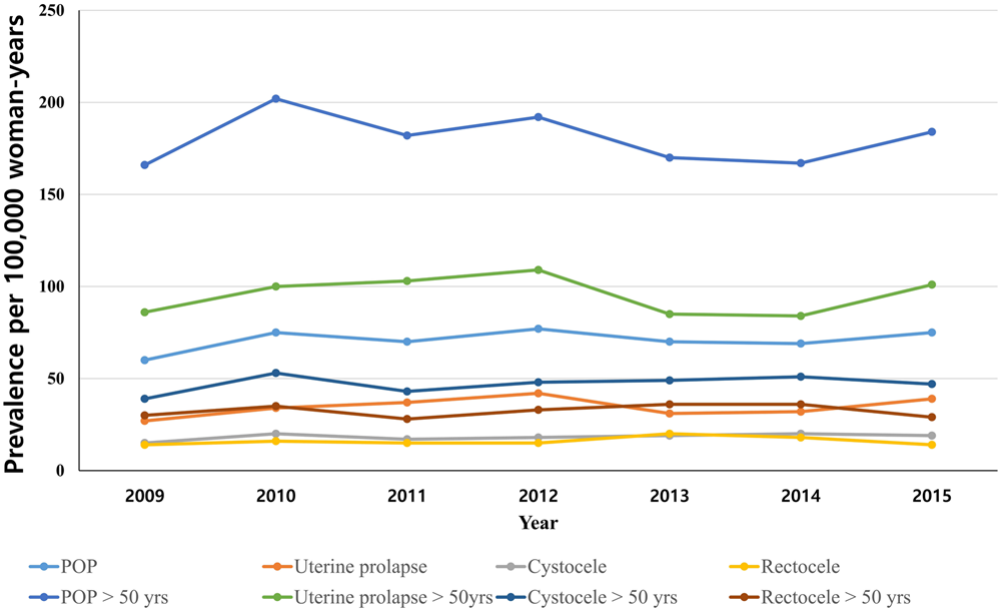
The trend in prevalence according to year increments in HIRA-NIS 2009–2015. HIRA-NIS: The Health Insurance Review & Assessment Service-National Inpatient Sample, POP: Pelvic Organ Prolapse.

The prevalence of POP disease did not significantly differ according to year (Fig. 2, Table 2). In logistic regression analysis after adjustment for age and year data (HIRA-NIS 2009–2015), constipation increased the prevalence of all POP disorders. In particular, constipation increased the prevalence of rectocele by 16.7-fold {women of all ages; odds ratio (OR), 16.66; 95% confidence interval (CI), 13.76–20.17; P < 0.01}(women greater than 50 years of age; OR, 16.67; CI, 13.04–21.33; P < 0.01) (Table 2).

**Table 2:** Logistic regression analysis in pelvic organ prolapse in HIRA-NIS 2009–2015.

		Women of all ages		Women aged over 50 years	
		OR (95% CI)	P-value	OR (95% CI)	P-value
POP	Age per 5 years	1.40 (1.38–1.41)	<0.01	1.24 (1.22–1.27)	<0.01
	Data year	0.99 (0.97–1.01)	0.24	0.99 (0.97–1.01)	0.44
	Low SES	0.76 (0.65–0.89)	<0.01	0.80 (0.68–0.95)	<0.01
	COPD	1.15 (0.81–1.64)	0.44	1.24 (0.87–1.78)	0.23
	Constipation	4.04 (3.52–4.63)	<0.01	3.71 (3.17–4.34)	<0.01
Uterine prolapse	Age per 5 years	1.53 (1.50–1.55)	<0.01	1.36 (1.32–1.39)	<0.01
	Data year	1.00 (0.97–1.02)	0.76	0.99 (0.97–1.02)	0.66
	Low SES	0.80 (0.64–0.99)	0.04	0.82 (0.66–1.03)	0.08
	COPD	1.41 (0.90–2.19)	0.13	1.50 (0.96–2.34)	0.07
	Constipation	1.85 (1.44–2.38)	<0.01	1.89 (1.46–2.44)	<0.01
Cystocele	Age per 5 years	1.40 (1.38–1.42)	<0.01	1.17 (1.14–1.21)	<0.01
	Data year	1.00 (0.97–1.03)	0.98	1.02 (0.98–1.05)	0.33
	Low SES	0.48 (0.40–0.58)	<0.01	0.52 (0.43–0.63)	<0.01
	COPD	0.54 (0.36–0.82)	<0.01	0.59 (0.39–0.91)	0.02
	Constipation	2.12 (1.53–2.94)	<0.01	2.35 (1.67–3.31)	<0.01
Rectocele	Age per 5 years	1.19 (1.17–1.21)	<0.01	0.97 (0.92–1.01)	0.16
	Data year	1.00 (0.97–1.04)	0.9	1.01 (0.96–1.05)	0.82
	Low SES	0.77 (0.56–1.07)	0.12	0.88 (0.59–1.31)	0.53
	COPD	1.02 (0.44–2.38)	0.96	1.26 (0.53–3.01)	0.6
	Constipation	16.66 (13.76–20.17)	<0.01	16.67 (13.04–21.33)	<0.01

COPD: Chronic Obstructive Pulmonary Disease, HIRA-NIS: The Health Insurance Review & Assessment Service-National Inpatient Sample, OR: Odd Ratio, POP: Pelvic Organ Prolapse, SES: Socioeconomic status.

Data year represents from HIRA-NIS 2009 to HIRA-NIS 2015.

Low socioeconomic status (SES) reduced the prevalence of cystocele (women of all ages; OR, 0.48; CI, 0.40–0.58; P < 0.01) (women greater than 50 years of age; OR, 0.52; CI, 0.43–0.63; P < 0.01) but did not affect the prevalence of uterine prolapse and rectocele (Table 2). The number of women requiring pessary only, surgery only, and any treatment excluding PFME were 9 ± 1 per 100,000 population, 36 ± 0 per 100,000 population, and 45 ± 1 per 100,000 population at all ages, respectively, and 26 ± 2 per 100,000 population, 89 ± 1 per 100,000 population, and 114 ± 2 per 100,000 population among women greater than 50 years of age, respectively.

**Figure 3:**
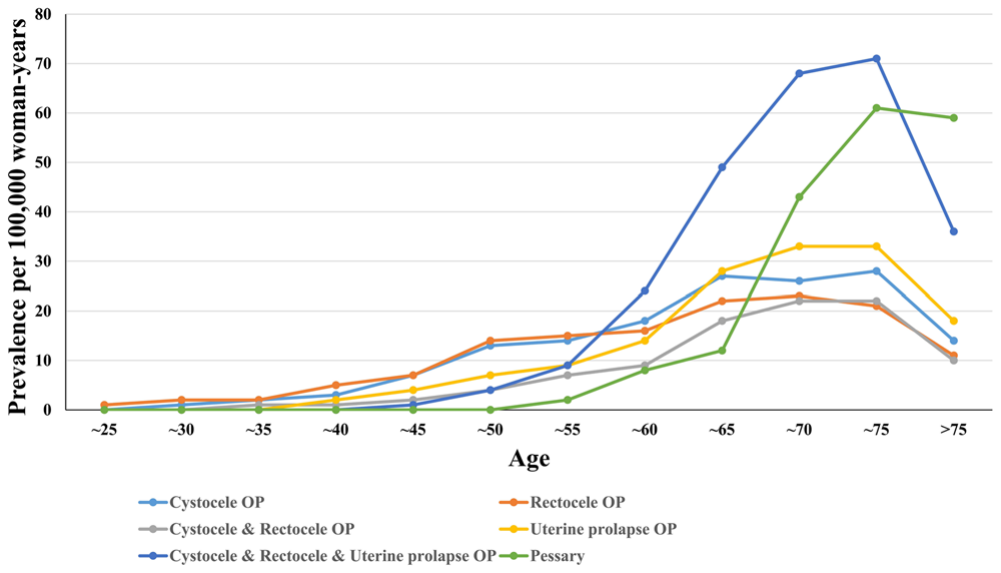
The change of pelvic organ prolapse treatment procedure according to 5-age increments in HIRA-NIS 2009–2015. OP: operation.

POP surgery was performed most often in women in their late 60 s and early 70 s, but the use of pessary was performed most often in women in their 70 s (Fig. 3). After 75 years of age, the use of pessary was higher than surgery. Of the total POP patients, 46% did not receive any special treatment, 44% underwent surgery, 9% used pessary, and 1% were treated with surgery and pessary.

## Discussion

In this study, the prevalence of POP was 71 ± 1 per 100,000 population for all ages and 180 ± 4 per 100,000 population for women older than 50 years old. This rate is much lower than the prevalence reported in previous studies (2.9–41.1%)^5–8^. This finding is attributed to differences in POP definitions. Hendrix *et al*. diagnosed POP through direct physical examination, whereas other studies diagnosed POP using a survey about symptoms^5–8^. However, in our study, POP for the prevalence calculation was defined the case was diagnosed by the doctor in clinic. POP patients diagnosed by physical examination may include patients without discomfort. POP patients diagnosed based on symptoms may not exhibit POP by pelvic examination. In fact, the prevalence in this study (0.07%) was much lower than the prevalence of symptom-based studies (2.9–8.3%)^6–8^. This finding indicates that the degree of discomfort to be treated is relatively low. The prevalence in this study is clinically more important than the prevalence reported by other studies because treating POP is determined by the degree of discomfort experienced by the patient^2^.

Our study reported that the prevalence of uterine prolapse was higher than that of cystocele. However, interpretation of this result requires caution. Our result represented exactly that the prevalence of diagnostic codes with uterine prolapse (Incomplete uterovaginal prolapse N81.2, Complete uterovaginal prolapse N81.3) was higher than that of diagnostic code with cystocele (Cystocele N81.1). However, cystocele or uterine prolapse is included in other diagnostic codes (Unspecified uterovaginal prolapse N81.4, Unspecified female genital prolapse N81.9). The ratios of cystocele or uterine prolapse in these codes are unknown. Therefore, this result should be interpreted with caution.

The rate of surgery in this study was 36 ± 0 per 100,000 population for all ages (89 ± 1 per 100,000 population for women greater than 50 years old), which was much lower than the rate of surgery (150 per 100,000 population) in the previous studies^11,12^. In addition, the rate of surgery in the previous studies (150 per 100,000 population) was greater compared with the prevalence of POP in this study (71 ± 1 per 100,000 population at all ages)^11,12^. This finding indicates that the prevalence of POP in previous studies is increased compared with the prevalence of our study. The exact reason is unknown, but we assumed the following information could explain this difference. First, the difference can result from a racial difference. Most of previous studies included Caucasians (81 ~ 97%) from the U.S.A., whereas the majority of our study population was Korean^11,12^. Therefore, white women may be at an increased risk for POP compared with Korean women. However, no significant difference was noted in the prevalence between Caucasians and Asians in previous studies^5,7^. Further studies adjusting environment, race, and country are needed. Second, economic differences between the two countries may explain the finding. The gross domestic product (GDP) for each person ($ 27,632) in South Korea is only 48% of the US GDP ($ 57,293) in 2015^13^. Due to the relatively low economic power of South Korea, patients with mild POP may have a tendency to receive less treatment. However, given that South Korea provides national health insurance, the surgery cost paid by the individual is relatively inexpensive. Therefore, it is unlikely the economic differences between the two countries cause the differences in POP prevalence. Third, there is a possible difference in the demographic composition between studies. The risks, such as parity, body weight, and constipation, may be different from those of previous studies^7^.

Pessary has few contraindication and is preferred for non-surgical treatment. Thus, 72% of US specialist clinicians choose pessary as the primary treatment for POP^14–17^. Pessary is successful in 53–76% of patients. However, the severity of POP, duration of use and type of pessary vary^18–20^. Despite the relatively high preference and success rate, few studies on how often pessary is used are available. In our study, pessary was used in 10% of all POP patients and 18.5% of all surgical or pessary treatments. In contrast, POP surgery was performed in 45% of all POP patients and peaked in patients approximately 70 years old. The use of pessary increases with age. In particular, the use of pessary in women 75 years or older was noted more often than any single surgery. (Fig. 3) The reason for this finding might be that the risk of surgery increases with age^21,22^. Considering that previous pelvic surgery is a risk for pessary failure and the reoperation rate of POP surgery was 29.2%, pessary should be used more often in patients with POP in their late 40 s to early 60 s^11,15^.

In our study, constipation was an important risk for POP. The results of previous studies are not consistent with this finding^5,7,23,24^. Hendrix *et al*. claimed that constipation is not a risk for POP, whereas most recent studies claimed that constipation is an important risk for POP^5,7,23,24^. Constipation might damage the pelvic floor (nerve and connective tissue) by increasing intra-abdominal pressure^7^. In support of this notion, one study reported that constipation in young adults caused POP^25^. However, in our study, the odds ratio of constipation in rectocele {OR 16.66 (13.76–20.17)} was significantly increased compared with the odds ratio of constipation in cystocele or uterine prolapse (1.85–2.12). Although constipation worsens POP, rectocele potentially caused constipation unlike cystocele or uterine prolapse. Bozkurt *et al*. reported that rectocele is a risk of constipation^26^. Both diseases (constipation and rectocele) are likely to exhibit negative synergy with each other. Further studies of the causal relationship between rectocele and constipation are needed.

Our study has some limits. First, our study could not distinguish vault prolapse from uterine prolapse. Given that the HIRA-National Inpatient Sample (HIRA-NIS) used in our study contains one-year sample data, we could not confirm the presence of hysterectomy prior to 1 year. Second, our data did not confirm the stage for cystocele and rectocele. Therefore, the prevalence according to stage was not confirmed. Third, our study included no data on parity or occupation. Therefore, our study could not adjust these factors. However, given that the primary purpose of our study was to determine the prevalence of POP, we did not experience problems in obtaining the prevalence of POP.

In conclusion, the prevalence of POP was 180 ± 4 per 100,000 population among women over 50 years old, which was quite lower than that noted in previous studies. Surgery peaked approximately 70 years old. The use of pessary has increased dramatically in women older than 65 years, and this procedure is the most commonly used treatment for women over 75 years old.

## Materials and Methods

### Study Settings and Participants

The Republic of Korea provides medical insurance service {(the National Health Insurance Corporation (NHIC)} to almost all Koreans living in the Republic of Korea^27^. Given that the NHIC offers medical insurance services for most diseases except for special cases, such as cosmetic surgery, it contains copious medical information, such as gender, age, low-income households group, diagnosis name, surgery name, and prescription history^27^. The Health Insurance Review & Assessment Service (HIRA) is an organization that evaluates the medical expenses charged by medical institutions in a neutral manner. The HIRA decides whether the costs are appropriate and suggests that the NHIC pay these costs. Therefore, the HIRA shares a significant portion of NHIC data^27^.

The HIRA-NIS is annual sample data using a stratifed randomized sampling method provided by the HIRA for medical research. The HIRA-NIS perform sampling in each year. Therefore, the sample members of each year are not the same. Per the extraction method, the HIRA-NIS extracts data from 13% of patients who were admitted during a one-year period and 1% of patients who were not admitted during a one-year period. This study used HIRA-NIS 2009–2015 (Serial keys; 2009–0066/2010-0084/2011-0063/2012-0058/2014-0068/2015-0057)^27^.

The surgical and treatment codes used were the medical care benefits of the health insurance 2016 edition, and the diagnostic code used was the Korean Standard Classification of Diseases, 7th Edition (KCD-7), which was modified from the International Statistical Classification of Diseases and Related Health Problems, 10^th^ edition (ICD-10).

As a first step for calculating prevalence, only data from women are extracted from HIRA-NIS 2009–2015. Women with POP are defined as having two or more same diagnostic codes for each POP (Female urethrocele N81.0, Cystocele N81.1, Incomplete uterovaginal prolapse N81.2, Complete uterovaginal prolapse N81.3, Unspecified uterovaginal prolapse N81.4, Vaginal enterocele N81.5, Rectocele N81.6, Other female genital prolapse N81.8, or Unspecified female genital prolapse N81.9). Women who were treated with POP were defined as having a POP diagnostic code and a treatment code (Repair of cystocele R3620, Anterior colporrhaphy R0408/R0409, Correction for rectocele Q3020, Posterior colporrhaphy R0410/R0411, Anterior & posterior colporrhaphy R0412/R0413, Abdominal hysterectomy R4145/R4146, Vaginal hysterectomy R4202, Manchester surgery R4204, Uterine suspension R4215, Abdominal colpopexy R4111, Vaginal colpopexy R4112, Vaginal hysterectomy with anterior & posterior colporrhaphy R4203, or Insertion of pessary R4113) at the same time. To determine the disease risk, women with chronic obstructive pulmonary disease (J44.x) and constipation (K59.0) were defined as having the disease when they had more than applicable diagnosis code. We have defined women who received the livelihood program as the low SES group.

### Statistical Analysis

All statistical analyses in this study were performed using statistical program R version 3.3.2 (The R Foundation for Statistical Computing, Vienna, Austria). All statistical calculations were performed using two-tailed tests and were assumed to be statistically significant if the p-value was less than 0.05. The weighted t-test was utilized for the mean comparison of continuous variables, and the chi-square test was utilized for the comparison of categorical variables. The weighted logistic regression method was utilized to calculate the risk of multiple variables.

### Ethics

Given that this study uses data anonymized by a third party, it is not subject to the Institutional Review Board (IRB) under the South Korea’s Bioethics and Safety Act.
